# Ethyl Pyruvate Reduces Systemic Leukocyte Activation via Caspase-1 and NF-κB After Blunt Chest Trauma and Haemorrhagic Shock

**DOI:** 10.3389/fmed.2020.562904

**Published:** 2020-10-02

**Authors:** Scott Dieteren, Niklas Franz, Kernt Köhler, Aleksander Nowak, Sabrina Ehnert, Alexey Surov, Marcus Krüger, Ingo Marzi, Nils Wagner, Borna Relja

**Affiliations:** ^1^Experimental Radiology, Department of Radiology and Nuclear Medicine, Otto von Guericke University, Magdeburg, Germany; ^2^Department of Trauma, Hand and Reconstructive Surgery, University Hospital of the Goethe University Frankfurt, Frankfurt, Germany; ^3^Institute of Veterinary Pathology, Justus Liebig University Giessen, Giessen, Germany; ^4^Department of Trauma and Reconstructive Surgery, BG Trauma Center Tuebingen, Siegfried Weller Research Institute, Eberhard Karls University Tuebingen, Tübingen, Germany; ^5^Department of Radiology and Nuclear Medicine, Otto von Guericke University, Magdeburg, Germany; ^6^Department of Microgravity and Translational Regenerative Medicine, Clinic for Plastic, Aesthetic and Hand Surgery, Otto von Guericke University, Magdeburg, Germany

**Keywords:** ethyl pyruvate, leukocytes, inflammation, NF-κB, trauma

## Abstract

**Background:** Blunt chest (thoracic) trauma (TxT) and haemorrhagic shock with subsequent resuscitation (H/R) induce strong systemic and local inflammatory response, which is closely associated with apoptotic cell loss and subsequently impaired organ function. The underlying mechanisms are not completely understood, therefore, the treatment of patients suffering from TxT+H/R is challenging. In our recent studies, we have demonstrated local anti-inflammatory effects of ethyl pyruvate (EtP) in lung and liver after TxT+H/R. Here, the therapeutic potential of a reperfusion regime with EtP on the early post-traumatic systemic inflammatory response and apoptotic changes after TxT followed by H/R were investigated.

**Methods:** Female Lewis rats underwent TxT followed by haemorrhagic shock (60 min). Resuscitation was performed with own blood transfusion and either lactated Ringers solution (LR) or LR supplemented with EtP (50 mg/kg). Sham group underwent the surgical procedures. After 2 h blood as well as lung and liver tissues were obtained for analyses. Systemic activation of neutrophils (expression of CD11b and CD62L), leukocyte phagocytosis, apoptosis (caspase-3/7 activation), pyroptosis (caspase-1 activation) and NF-κB p65 activity were assessed. *p* < 0.05 was considered significant.

**Results:** TxT+H/R-induced systemic activation of neutrophils (increased CD11b and reduced CD62L expression) was significantly reduced by EtP. Trauma-induced delayed neutrophil apoptosis was further reduced by EtP reperfusion but remained unaltered in monocytes. Reperfusion with EtP significantly increased the phagocytizing capacity of granulocytes. Trauma-induced inflammasome activation, which was observed in monocytes and not in neutrophils, was significantly reduced by EtP in both cell entities. NF-κB p65 activation, which was increased in neutrophils and monocytes was significantly decreased in monocytes.

**Conclusion:** TxT+H/R-induced systemic activation of both neutrophils and monocytes concomitant with increased systemic inflammation was reduced by a reperfusion with EtP and was associated with a down-regulation of NF-κB p65 activation.

## Introduction

Trauma is the sixth leading cause of death and the leading cause of mortality in individuals under 35 years of age (1). Notably patients with blunt chest trauma, severe traumatic brain injuries (56.6%) and/or bleeding (18.5%) are at high risk for mortality; of which the last is responsible for 80% of all deaths that occur within the first hour (1). Patients who initially survive their injuries suffer from a dysfunctional post-traumatic inflammatory response with excessive systemic and local leukocyte activation which leads to tissues damage, acute lung and liver injury, multiple organ failure (MOF) and delayed mortality (2–4). MOF following haemorrhagic shock and resuscitation extends the intensive care and still remains one of the most significant contributors to late post-injury mortality (5).

Due to post-injury hypoperfusion with concomitant microcirculatory disturbances, endothelial damage, and tissue injury induce an extensive release of Pathogen-associated molecular patterns (PAMP) and/or Damage-associated molecular patterns (DAMP) (4, 6). Those stimulate, activate and recruit effector cells of the innate immune system e.g., granulocytes and monocytes to the sites of injury, which exert their defense strategies i.e. production, activation and/or release of proinflammatory mediators, proteases (neutrophil elastase), or reactive oxygen species (ROS) (3, 4, 6). During the post-traumatic priming of those cells, their phenotypic and functional shifts and changes occur, including the activation of adhesion molecules and diminished apoptosis (7). Trauma-associated activation of circulating granulocytes with a significant increase in CD11b expression (8, 9), which is promoting the adhesion of activated neutrophils to inflamed endothelia, and a decrease in CD62L expression, which plays an important role in tethering and rolling along postcapillary venules (10) has been shown. The increased pulmonary infiltration with activated polymorphonuclear leukocytes (PMNL) initiates the early state of post-traumatic acute respiratory distress syndrome (11, 12). Concomitant trauma is associated with delayed apoptosis of systemic neutrophils, which is mediated by Caspase-3/7, thus lowered apoptosis rates extending their live span (8, 13, 14). Maianski et al. demonstrated that apoptosis in neutrophils is linked to caspase-3 activation (15). Furthermore, the decreased apoptosis of circulating neutrophils persisted until 9 days after injury in traumatized patients (14). On the other hand, increasing neutrophil apoptosis early after trauma-haemorrhagic shock as well as inhibiting caspase-3 dependent apoptosis after shock resulted in tissue protective effects and protection from infection and organ failure (16, 17). Thus, this alteration suggests a prolonged presence of PMNL in tissues which may exaggerate the local post-traumatic proinflammatory response and cause tissue damage (13, 18). Conflictive data are provided regarding systemic activity of monocytes, which on the one hand is diminished by reduced inflammasome activation with caspase-1 as its key player in trauma patients (19, 20), and on the other hand an increase due to trauma and shock-induced pyroptosis in circulating monocytes (21, 22). Inflammasome activation and subsequent proinflammatory cell death so-called pyroptosis involve the activation of NF-κB, which is a central contributor in the production of proinflammatory cytokines, leukocyte recruitment, and cell survival (18, 23, 24). Oxidative burst in trauma-primed neutrophils correlate with elevated NF-κB p65 phosphorylation mediating the inflammatory response and tissue damage as well (24).

Ethyl pyruvate (EtP) is a stable ester formed from ethanol linked to pyruvate, which has demonstrated anti-inflammatory potential with tissue protective effects in several *in vivo* models of acute inflammation (25–27). Ethyl pyruvate ameliorated the inflammatory and apoptotic effects of cytokine- as well as trauma-induced inflammation in lung tissue and lung cells *in vivo* and *in vitro* (28–32). Its anti-inflammatory influence was associated with induced activation of the inflammasome, thus reduced caspase-1 activity and lower levels of interleukin (IL)-1β in endotoxin-primed macrophages (33). In ischemia models, a resuscitation with EtP reduced the local proinflammatory tumor necrosis factor (TNF) expression in liver, and improved survival by reducing mucosal hyperpermeability (34–36). In our recent studies, we have demonstrated tissue-protective effects of EtP with diminished levels of proinflammatory cytokines and reduced NF-κB p65 phosphorylation in lung and liver as well as reduced neutrophilic infiltration into those organs (32, 37). Thus, we studied the influence of a reperfusion regime with EtP on systemic inflammatory and apoptotic changes in leukocytes in our clinically relevant double-hit model of hemorrhage and blunt chest trauma.

## Materials and Methods

### Animals and Experimental Model

This study was approved by the veterinary department of the regional council in Darmstadt, Germany (Hessen, Germany; “Regierungspraesidium Darmstadt, Veterinaerswesen,” Hessen, Germany; Nr. of the ethical approval: FK/1028) and assigned in accordance with the ARRIVE guidelines (38). Only members with the certificate of the Federation of European Laboratory Animal Science Association treated and handed the animals. In the experiments, female LEWIS rats (190–240 g, Janvier Labs, France) were anesthetized with isoflurane (1.2–3.0%), buprenorphine (0.05 mg/kg body weight), and local anesthesia (0.25% Carbostesin) were applied. In detail, during anesthesia initiation (mask anesthesia), a 3.0% isoflurane oxygen mixture was used. The vessels were cannulated with a 1.2–2.5% isoflurane oxygen mixture depending on individual responses to pain stimulation. Trauma was performed with a concentration of 2% isoflurane following a short stabilization period and in the reperfusion period. At the end of experimentation, the isoflurane concentration was reduced in a step-by-step manner. The animals breathed spontaneously and were not intubated. The abdomen, chest, right inguinal and the neck of the animals were shaved and marked for surgical preparation. The right femoral artery was cannulated with polyethylene tubing for continuous blood pressure measurement. For the bilateral lung contusion the animals placed and a standardized air blast wave was directed to the thorax (TxT) of the animals as described before (39). After a short stabilization phase, the left jugular vein and the right carotid artery were cannulated. Then haemorrhagic shock (HS) by withdrawing stepwise blood via the carotid artery until a mean arterial blood pressure (MABP) of 35 ± 3 mm Hg was initiated. Over a period of 60 min the MABP was monitored by a blood pressure analyzer (Siemens AG), and if necessary was kept constant by further withdrawal or recirculation of withdrawn blood (40, 41). At the end of the haemorrhagic shock, reperfusion (H/R) was carried out via the jugular vein. Resuscitation was either performed by reperfusion with 60% of the shed blood plus 50% Ringer's lactated solution (LR) or 60% blood plus 50% LR supplemented with EtP (Sigma Aldrich, 50 mg/kg body weight). After an observation and stabilization phase of 30 min the catheters were removed, the vessels were occluded and the wounds closed. A continuous temperature monitoring in the colon maintaining 37°C was carried out. The animals always had free access to food and water. Two hours after the end of experiments sampling was performed. Briefly, the sacrifice was performed by withdrawing blood *via* the aorta using the same isoflurane oxygen concentration as during the preparation period and flushing the organs with LR.

### Group Allocation

Thirty animals were randomly assigned to sham, TxT+H/R_LR and TxT+H/R_EtP groups. The sham groups underwent all the surgical procedures without inducing TxT+H/R. The trauma groups were resuscitated either with LR solution or LR solution supplemented with EtP.

### Measurement of Antigen Expression by Flow Cytometry

**Figure 1:**
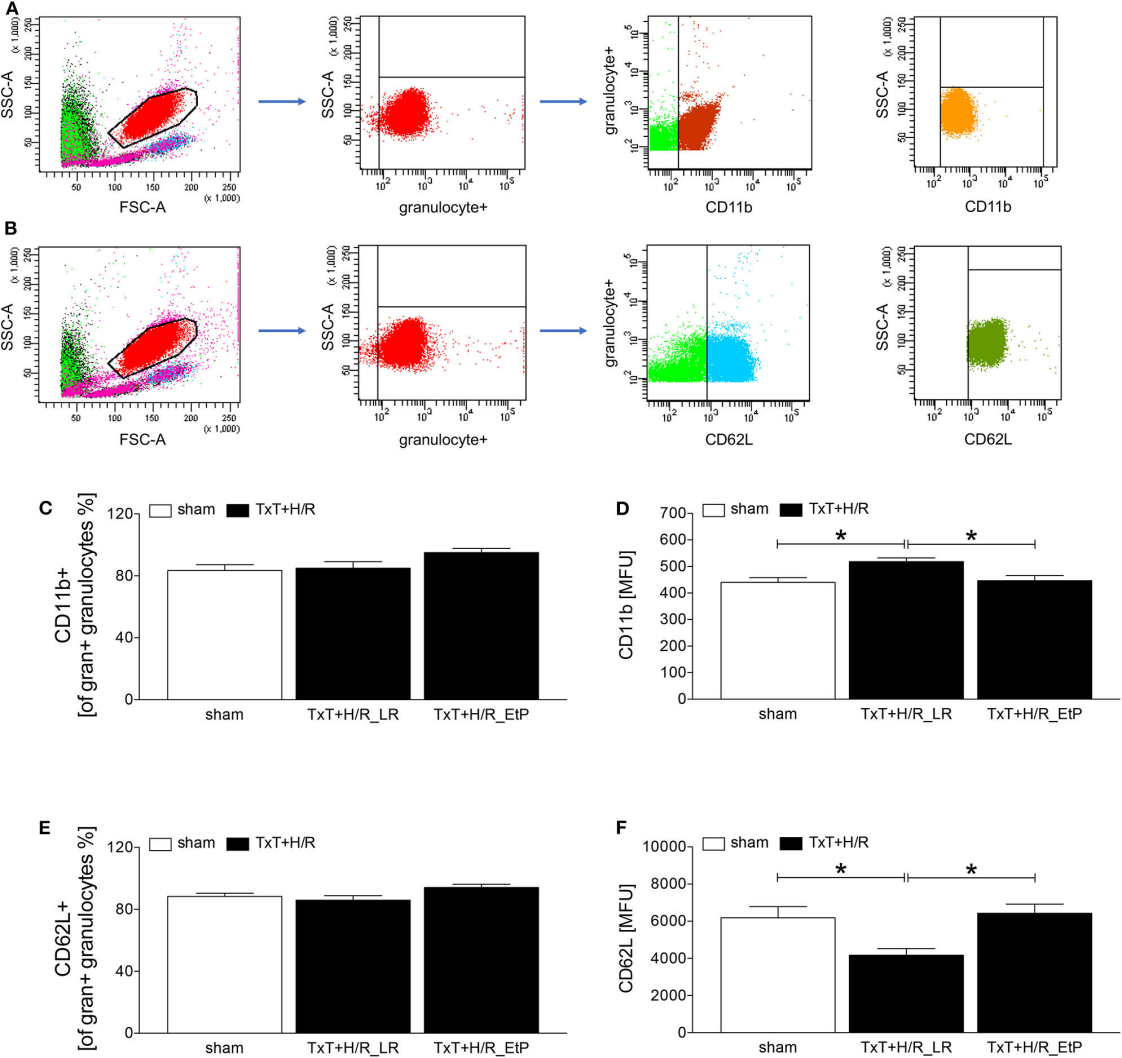
Flow cytometric analysis of cellular adhesion molecule CD11b and CD62L on circulating neutrophils after blunt thoracic trauma (TxT) followed by haemorrhagic shock and resuscitation (H/R). Two hours after resuscitation flow cytometric analysis of CD11b or CD62L expression on circulating granulocytes **(A-F)** was performed. Sham operated animals underwent all surgical procedures without induction of TxT and H/R. TxT+H/R_LR animals received lactated Ringers solution (LR) and TxT+H/R_EtP animals were resuscitated with LR supplemented with ethyl pyruvate (EtP). **(A)** Gating strategy for CD11b positive granulocyte. **(B)** Gating strategy for CD62L positive granulocytes. **(C)** Percentage of CD11b^+^ cells out of all gran^+^ granulocytes. **(D)** Mean fluorescence units (MFU) of CD11b^+^ on gran^+^ granulocytes. **(E)** Percentage of CD62L^+^ cells out of all gran^+^ granulocytes. **(F)** MFU of CD62L^+^ on gran^+^ granulocytes. Data are given as mean ± standard error of the mean, *p < 0.05 vs. indicated, *n* = 10.

Blood samples were collected from the aorta in pyrogen-free heparinized tubes for cytometric flow analyses. Blood samples (50 μl) were transferred into polystyrene fluorescence-activated cell sorter (FACS) tubes (BD Pharmingen™) and incubated with mouse anti-rat Granulocytes (RP-1, gran^+^) (Bio Legend, conjugated to Mix-n-Stain CF405 Antibody Labeling Kit from Sigma-Aldrich), anti-rat CD62L allophycocyanin (APC) (Bio Legend), and mouse anti-rat CD11b fluorescein isothiocyanate (BD Pharmingen™) antibodies. For the settings, control stainings with the corresponding isotype antibodies were applied. After 30 min at room temperature (RT) one ml of the FACS Lysing Solution (BD Pharmingen™) was added for additional 10 min (RT). Then samples were centrifuged at 400 g for 7 min and washed twice with two ml FACS buffer. After removal of the supernatants, cells were diluted in 400 μl FACS buffer and subjected to flow cytometric analyses. Either granulocytes or monocytes were defined by gating the corresponding forward and side scatter scan. The granulocyte population was additionally gated by the RP-1 positive cells in the corresponding forward and sideward scatter scan (Figures 1A,B). From each sample a minimum of 30,000 cells was measured. The percentage of positive cells and the MFU were determined.

### Phagocytosis of Staphylococcus Aureus Bioparticles

A single vial of lyophilized pHrodo *Staphylococcus* (*S*.) *aureus* Red BioParticles Conjugate (Life Technologies, Paisley, UK) was resuspended as suggested by the provider, and the suspension was added to 100 μl of whole blood and treated according to the manufacturer's instructions for 1 h at 37°C and 5% CO_2_. A negative control without BioParticles was included. Then, red blood cells were removed using FACS Lysing Solution diluted with distilled water (1:10, Biosciences, Heidelberg, Germany) for 10 min. Two ml phosphate-buffered saline (PBS) were added and the cells were centrifuged at 400 g for 7 min at room temperature. Thereafter, the cells were washed with 3 ml FACS buffer (PBS w/o Ca${\;}_{2}^{+}$/Mg^2+^ supplemented with 0.5% bovine serum albumin). After the centrifugation, the supernatants were removed and the cells were diluted in 400 μl FACS buffer. The phagocytizing activity of granulocytes and monocytes, respectively, was quantified as a percentage of phagocytizing cells as well as their capacity by determining the mean fluorescence units (MFU) using a BD FACS Canto 2™ and FACS DIVA™ software (BD). Either granulocytes or monocytes were defined by gating the corresponding forward and side scatter scan. From each sample a minimum of 30,000 cells was measured.

### Caspase-3/7 and Caspase-1 Activation Assay

Active caspases were quantified by using a FAM-DEVD-FLICA caspase-3/7 kit and FAM-YVAD-FMK 660 caspase-1 detection kit (ImmunoChemistry Technologies) according to the manufacturer's guidelines. MFU of the cells were quantified by flow cytometry (using unstained cells to set the gate) by BD FACS Canto 2™ and FACS DIVA™ software. Each cell population was defined by gating the corresponding forward and side scatter scan.

### Measurement of NF-κB p65 (Phospho) Expression by Flow Cytometry

Blood samples (50 μl) were transferred into polystyrene FACS tubes (BD Pharmingen™) and incubated with mouse anti-rat Granulocytes (RP-1) (Bio Legend, conjugated to Mix-n-Stain CF405 Antibody Labeling Kit from Sigma-Aldrich), and mouse anti-rat CD68 Alexa Fluor 700 (Abd Serotec) antibodies. After 30 min at RT the samples were washed with 4 ml FACS buffer. Then, supernatants were removed and the samples were incubated with 100 μl Fix & Perm Solution A (FIX&PERM Kit, An Der Grub) for 15 min at RT. After another washing procedure with PBS, the supernatants were removed and samples were incubated with 100 μl Fix & Perm Solution B, and anti NF-κB p65 (Abcam, conjugated with APC/Cy7 Conjugation Kit from Abcam) anti phospho NF-κB p65/Ser536 (Abcam, conjugated with Mix-n-Stain CF488A Antibody Labeling Kit from Sigma-Aldrich) antibodies for 30 min at RT. Subsequently, 2 ml of FACS lysing solution (FACS Lysing Solution, BD Pharmingen™) were added for additional 10 min and the samples were centrifuged at 400 g for 5 min. Another washing procedure with 4 ml FACS buffer followed. After removal of the supernatants, cells were diluted in 400 μl FACS buffer and subjected to flow cytometric analyses and gated as described above.

### Lung and Liver Preparation

After collecting blood samples flushing with 20 ml LR followed. *Via* the portal vein 10% buffered formalin solution was infused as it was done into the lung lobe itself. Then, the samples were embedded in paraffin and subsequently sectioned and stained with hematoxylin-eosin (H&E). The histological examination of the tissue morphology in both organs was performed by an independent pathologist who allocated the H&E-stained sections to the different experimental groups in a blinded manner as described before (32, 37).

### Statistical Analysis

Normality distribution was assessed by Kolmogorov-Smirnov test with Dallal-Wilkinson-Lilliefor *P*-value. Non-parametric Kruskal-Wallis test was applied to study the differences between the groups. For *post-hoc* corrections Dunn's multiple comparison test was applied. Data are given as mean ± standard error of the mean (sem). A *p*-value below 0.05 was considered statistically significant. All statistical analyses were performed using GraphPad Prism 6 (Graphpad Software, Inc., San Diego, CA).

## Results

### Granulocyte Activity After Blunt Thoracic Trauma Followed by Hemorrhage and Resuscitation

Two hours after resuscitation CD11 and CD62L expression levels on granulocytes were assessed to evaluate the impact of EtP on neutrophil activation after TxT+H/R (Figures 1A–F). The proportion of CD11b positive of all granulocyte^+^ gated granulocytes was not significantly changed between the groups, however, a trend to an increase was observed in the TxT+H/R_EtP group (Figure 1C). CD11b expression on granulocytes was significantly increased in TxT+H/R_LR group as compared to the sham group (518.10 ± 14.36 vs. 440.00 ± 318.19 MFU, respectively, *p* < 0.05, Figure 1D). Resuscitation with EtP diminished CD11b expression significantly in relation to the TxT+H/R_LR group (446.40 ± 19.19 vs. 518.10 ± 14.36 MFU, respectively, *p* < 0.05, Figure 1D). CD11b expression levels in the EtP group were comparable to those in the sham group (*p* < 0.05, Figure 1D).

Similar to the CD11b data, the proportion of CD62L positive of all granulocyte^+^ gated granulocytes exerted a trend to an increase in the TxT+H/R_EtP group, however, this was not significant (Figure 1E). CD62L expression significantly decreased in TxT+H/R_LR group compared to the sham group (4174.00 ± 355.80 vs. 6186.00 ± 599.70 MFU, respectively, *p* < 0.05, Figure 1F). Resuscitation with EtP significantly increased the CD62L expression on granulocytes compared to the TxT+H/R_LR group (6427.00 ± 497.60 vs. 4174.00 ± 355.80 MFU, respectively, *p* < 0.05, Figure 1F). The CD62L expression levels in the EtP group were comparable to the sham group.

### Systemic Leukocyte Phagocytosis After Blunt Thoracic Trauma Followed by Hemorrhage and Resuscitation

**Figure 2:**
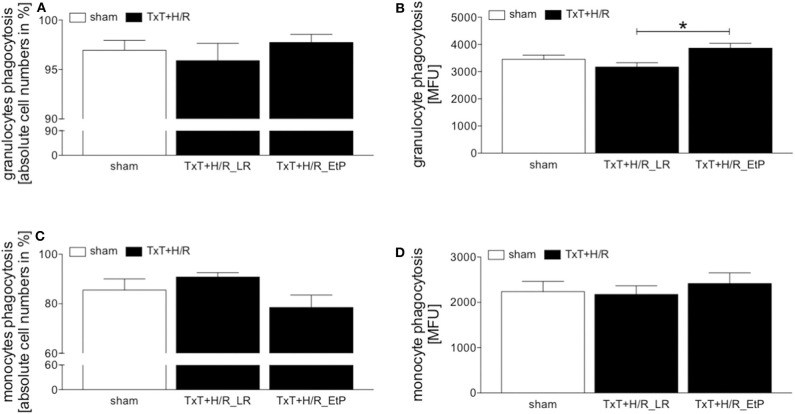
Flow cytometric analysis of granulocyte and monocyte phagocytosis after blunt thoracic trauma (TxT) followed by haemorrhagic shock and resuscitation (H/R). Two hours after resuscitation flow cytometric analysis of phagocytosis in circulating granulocytes and monocytes was performed. Sham operated animals underwent all surgical procedures without induction of TxT and H/R. TxT+H/R_LR animals received lactated Ringers solution (LR) and TxT+H/R_EtP animals were resuscitated with LR supplemented with ethyl pyruvate (EtP). **(A)** Percentage of phagocytizing granulocytes in absolute cell numbers. **(B)** Mean fluorescence units (MFU) of phagocytizing granulocytes. **(C)** Percentage of phagocytizing monocytes in absolute cell numbers. **(D)** MFU of phagocytizing monocytes. Data are given as mean ± standard error of the mean, *p < 0.05 vs. indicated, *n* = 10.

The proportion of phagocytizing granulocytes did not show significant changes (Figure 2A). However, the phagocytizing capacity was significantly reduced in the TxT+H/R_LR vs. TxT+H/R_EtP group (3169.00 ± 156.10 vs. 3864.00 ± 175.00 MFU, *p* < 0.05, Figure 2B). Comparing the proportion of phagocytizing monocytes, there were no significant changes between the groups, however, some trends were observed (Figures 2C,D).

### Systemic Leukocyte Apoptosis After Blunt Thoracic Trauma Followed by Hemorrhage and Resuscitation

**Figure 3:**
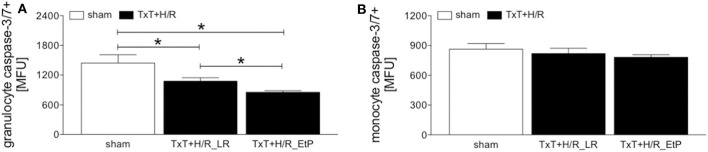
Caspase-3/7 activation after blunt thoracic trauma (TxT) followed by haemorrhagic shock and resuscitation (H/R) in granulocytes and monocytes. Two hours after resuscitation flow cytometric analysis of apoptosis (caspase-3/7 activation) in circulating granulocytes **(A)** and monocytes **(B)** was performed. Sham operated animals underwent all surgical procedures without induction of TxT and H/R. TxT+H/R_LR animals received lactated Ringers solution (LR) and TxT+H/R_EtP animals were resuscitated with LR supplemented with ethyl pyruvate (EtP). **(A)** Mean fluorescence units (MFU) of caspase-3/7 positive granulocytes and **(B)** monocytes. Data are given as mean ± standard error of the mean, *p < 0.05 vs. indicated, *n* = 10.

Monocyte and granulocyte apoptosis were assessed by measuring the level of activated caspase-3/7 2 h after resuscitation (Figures 3A,B). In granulocytes TxT+H/R significantly reduced caspase-3/7 activation in the LR group compared to the sham group (1076.00 ± 70.79 vs. 1445.00 ± 168.30 MFU, *p* < 0.05, Figure 3A). Moreover, resuscitation with EtP significantly diminished caspase-3/7 activation in granulocytes after TxT+H/R compared to both TxT+H/R_LR as well as to the sham group (854.40 ± 30.78 vs. 1076.00 ± 70.79 and 1445.00 ± 168.30 MFU, respectively, *p* < 0.05, Figure 3A).

In monocytes, no significant changes in caspase-3/7 activation were detected between the groups (Figure 3B).

### Systemic Leukocyte Pyroptosis After Blunt Thoracic Trauma Followed by Hemorrhage and Resuscitation

**Figure 4:**
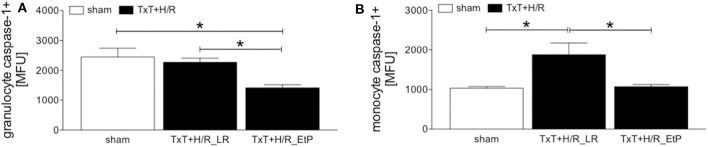
Caspase-1 activation after blunt thoracic trauma (TxT) followed by haemorrhagic shock and resuscitation (H/R) in granulocytes and monocytes. Two hours after resuscitation flow cytometric analysis of pyroptosis (caspase-1 activation) in circulating granulocytes **(A)** and monocytes **(B)** was performed. Sham operated animals underwent all surgical procedures without induction of TxT and H/R. TxT+H/R_LR animals received lactated Ringers solution (LR) and TxT+H/R_EtP animals were resuscitated with LR supplemented with ethyl pyruvate (EtP). **(A)** Mean fluorescence units (MFU) of caspase-1 positive granulocytes and **(B)** monocytes. Data are given as mean ± standard error of the mean, *p < 0.05 vs. indicated, *n* = 10.

Monocyte and granulocyte pyroptosis were assessed by measuring the activity of caspase-1 2 h after resuscitation (Figures 4A,B). In granulocytes, no significant changes were observed between the sham and TxT+H/R_LR group. However, there was a significant decrease in pyroptosis in the TxT+H/R_EtP group compared to both sham and TxT+H/R_LR (1414.00 ± 102.60 vs. 2444.00 ± 293.00 and 2267.00 ± 137.00 MFU, respectively, *p* < 0.05, Figure 4A).

In monocytes caspase-1 activity was significantly increased after TxT+H/R compared to the sham group (1880.00 ± 296.20 vs. 1033.00 ± 44.22, *p* < 0.05, Figure 4B). TxT+H/R followed by resuscitation with EtP significantly decreased the caspase-1 activation compared with the TxT+H/R_LR group to the levels comparable to those of the sham animals (1074.00 ± 52.12 vs. 1880.00 ± 296.20, *p* < 0.05, Figure 4B).

### Analysis of NF-κB p65 Phosphorylation in Systemic Leukocytes After Blunt Thoracic Trauma Followed by Hemorrhage and Resuscitation

**Figure 5:**
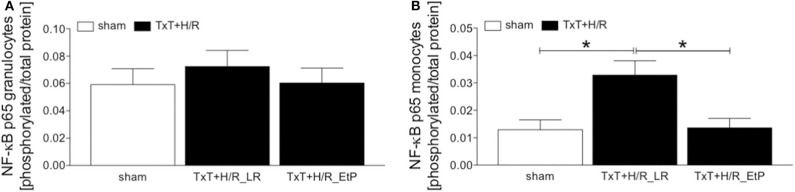
Flow cytometric analysis of NF-κB p65 phosphorylation after blunt thoracic trauma (TxT) followed by haemorrhagic shock and resuscitation (H/R). Two hours after resuscitation flow cytometric analysis of phosphorylated NF-κB p65 in circulating granulocytes **(A)** and monocytes **(B)** was performed. Sham operated animals underwent all surgical procedures without induction of TxT and H/R. TxT+H/R_LR animals received lactated Ringers solution (LR) and TxT+H/R_EtP animals were resuscitated with LR supplemented with ethyl pyruvate (EtP). The ratio of phosphorylated to total NF-κB protein in granulocytes **(A,B)** monocytes is given. Data are given as mean ± standard error of the mean, *p < 0.05 vs. indicated, *n* = 10.

The ratio of the expression of NF-κB p65 phosphorylated protein to the total p65 protein in granulocytes and monocytes 2 h after resuscitation was evaluated (Figures 5A,B). There were no significant changes in the ratio of NF-κB p65 phosphorylated/total NF-κB p65 protein, however, there was a trend to an increase after TxT+H/R in the LR group compared with both sham as well as TxT+H/R_EtP groups in granulocytes (Figure 5A).

TxT+H/R_LR induced a significant increase in the expression of NF-κB p65 phosphorylated/total NF-κB p65 protein in monocytes compared to sham as well as to the TxT+H/R_EtP group (*p* < 0.05, Figure 5B).

### Histomorphological Changes in Lung and Liver

Both lung and liver showed prominent organ damage in the TxT+H/R_LR group (data not shown). In lungs, alveolar wall thickening with disruption and cellular infiltrates compared to the sham groups were detected. TxT+H/R-induced changes were markedly reduced in the TxT+H/R_EtP group. Liver sections from the TxT+H/R_LR group revealed large areas of necrosis compared to the sham group (data not shown). TxT+H/R-induced changes were markedly reduced in the TxT+H/R_EtP group with less necrotic areas in liver sections. No tissue damage or necrosis were observed in the sham group. The representative and detailed analyses of the local effects of a EtP-reperfusion on lungs and liver in the underlying model of TxT and H/R are shown in our referred studies (32, 37).

## Discussion

In our previous studies, we have shown significant local anti-inflammatory and organ-protective effects of ethyl pyruvate after trauma (32, 37), which has been confirmed as a safe drug to use in cardiac surgery in a phase II multicentre study (42). Here, we investigated if the reperfusion with EtP will affect systemic inflammatory response in leukocytes induced by a double-hit model of blunt chest trauma and haemorrhagic shock. The anti-inflammatory potential of EtP is associated with reduced systemic inflammation of neutrophils and local organ damage as shown before in *in vitro* and *in vivo* studies (43, 44). Endotoxin-induced acute lung injury was attenuated by EtP due to the inhibition of cytokine production of IL-6 and TNF-α (44). However, although there are diverse studies demonstrating anti-inflammatory effects of EtP, the applied models do not mimic often occurring clinical situation with traumatic insult of blunt chest trauma concomitant with haemorrhagic shock, as it has been applied in the underlying study.

Trauma-induced release of PAMPs and/or DAMPs into circulation primes systemic neutrophils inciting non-specific organ damage (3, 45). Those activated circulating cells may cause dysfunctions of endothelial barriers due to increased migratory and functional capacity with enhanced release of ROS and elastases which damage the endothelia in critically ill trauma patients. Samples from traumatized patients who develop pneumonia show increased migratory potential of circulating neutrophils *via* elevated Mac-1 and reduced L-selectin levels (46). CD11b and CD62L play an important role in tethering and rolling along endothelia and transmigration into sites of inflammation (8, 47). During neutrophil activation, rolling and transmigration to sites of inflammation, CD62L can be shedded as soluble L-selectin from the cell surface into the circulation (48, 49). In our study, we also show upregulated CD11b and reduced CD62L levels on circulating neutrophils, indicating at an enhanced activation of neutrophil after trauma and hemorrhage in our model. In line with our findings Visser et al. have shown that the transient activation of neutrophils and mobilization of young neutrophils into the circulation was accompanied by decreased CD62L expression in a model of blunt chest injury (10). According to Mommsen et al. CD62L shedding from the surface of neutrophils may be a protective mechanism against inflammation after surgical trauma (50). The elevated levels of soluble L-selectin may interfere with L-selectin-mediated migration by competing for ligand binding, resulting in decreased leucocyte delivery to sites of inflammation (51, 52). In contrast to this, CD62L shedding is proposed to modulate the ability of leukocytes to migrate and enter sites of inflammation (51). Thus, CD62L shedding is required for activated leukocytes to detach from endothelia before their extravasation into tissues (51). This is in line with our findings showing decreased CD62L levels on circulating neutrophils after TxT+H/R. Adjacent to CD62, CD11b is responsible for mediating neutrophil adhesion to vascular endothelia (8). Traumatic brain injury was accompanied by significantly elevated CD11b expression on circulating neutrophils and increased neutrophil infiltration into tissue after injury (53). In line with these reports, the double-hit as applied in our model induced CD11b. Moreover, the combination of decreased CD62L and increased CD11b expression on circulating neutrophils after trauma is in line with results of Hazeldine et al. (54). With regard to monocytes, in traumatized patients with sepsis and acute respiratory distress syndrome a similar systemic activation of peripheral blood mononuclear cells as well as their pyroptosis and apoptosis compared to those patients without complications has been shown (55). In terms of functional analyses, next to their activation, trauma induces modulations in neutrophils such as increased life-span which is caused by lowered apoptosis rates (8, 14). Interestingly, it has been shown that decreased apoptosis in circulating neutrophils persisted until 9 days after injury in trauma patients, contributing to organ dysfunction due to prolonged neutrophil hyperactivity (14). Maianski et al. demonstrated that apoptosis in neutrophils is linked to caspase-3 activation (15).The inhibited neutrophil apoptosis and prolonged live span promoted local inflammation in lung tissue (56). The data are in line with our observations demonstrating reduced caspase-3/7 activation after double-hit trauma and showing prolonged live span in our model. Anne Morrison et al. indicated that early increased neutrophil apoptosis in trauma-haemorrhagic shock may prevent from developing subsequent infection and MOF (16). Similarly, in a rat model of haemorrhagic shock, inhibition of caspase-3 dependent apoptosis resulted in tissue protective effects (17). Furthermore, major trauma leads to rapid recruitment of circulating monocytes, that serve as phagocytizing cells and executors of tissue healing and of an effective inflammatory response (57). Here, notably pyroptosis, a specific form of inflammasome- and inflammation-induced cell death can amplify the inflammatory response (58). Inflammasomes are promoting inflammation by processing and thus activation of proinflammatory cytokines notably IL-1β or IL-18 *via* i.e., caspase-1 (59). Our data show increased pyroptosis *via* elevated caspase-1 levels in circulating monocytes after TxT+H/R indicating at trauma-induced proinflammatory effects on circulating monocytes. In terms of pathomechanistical pathways, Nolan has shown that reduced apoptosis in systemic neutrophils after trauma may be NF-κB dependent (18). NF-κB plays a central role in the initiation and regulation of systemic and local immune response after trauma (23, 60, 61). In our study, TxT+H/R significantly increased NF-κB p65 phosphorylation in circulating monocytes. This is in line with increased NF-κB activity in monocytes of multiply injured trauma patients in the early post-traumatic phase (61, 62).

Our results and data from others indicate that organ-protective effects of EtP may be caused by a decreased systemic activation of leukocytes. Decreased CD62L levels on circulating neutrophils after TxT+H/R were significantly increased by EtP. Thus, the data suggest, that TxT+H/R-induced systemic activation can be diminished by EtP, subsequently reducing the transmigration of neutrophils to sites of inflammation upon a traumatic insult. Moreover, in a model of *E. coli*-induced sepsis, treatment with EtP significantly diminished inflammation by reduced systemic leukocyte rolling, adherence and migration in the mesenteric microcirculation (63). In the present study, resuscitation with EtP reduced the expression of CD11b on circulating neutrophils, indicating at an anti-inflammatory effect *via* inhibition of systemic activation of leukocytes. Intriguing data have been found after resuscitation with EtP which further reduced caspase-3/7 activity in circulating granulocytes, effects that can be associated with diminished tissue damage in liver and lung as shown in our previous studies (32, 37). This is in contrast to above described findings, where tissue-protective effects were demonstrated by restoring apoptotic capacity in neutrophils. Tissue-protective effects of EtP are potentially caused by a diminished ability of neutrophils to transmigrate into sites of inflammation and additionally inhibiting apoptosis *via* caspase-3 in tissues. This is in line with the results of Sharma et al. in a model of haemorrhagic shock, where liver protective by an EtP resuscitation were linked to the inhibition of caspase-3 induced cell apoptosis (17). In our study resuscitation with EtP had no significant effect on apoptosis of circulating monocytes. Resuscitation with EtP significantly diminished the inflammasome activity in monocytes to levels comparable to those of the untreated sham group. This is in line with Li et al. who has shown that EtP reduced IL-1β expression in macrophages *via* inhibiting caspase-1 activation through inflammasome (33). Interestingly, also in neutrophils EtP reduced the caspase-1 activity to levels below sham and trauma groups, data which might indicate at a general effect of EtP on pyroptosis in circulating immune cells. Furthermore, Yang et al. provided the first clear evidence that EtP exerts its anti-inflammatory effects *via* decreasing the activation of NF-κB, which is then followed by a downregulated expression of proinflammatory genes in liver and colon mucosa (36). In an *in vitro* experiment with macrophage like RAW and kidney cells, EtP directly targets the p65 subunit of the transcription factor, modifying the cysteine 38 and inhibiting NF-κB signaling (64). In a model of traumatic brain injury, multiple injections with EtP significantly inhibited NF-κB p65 presence in the nucleus of damaged microglia cells, improving blood brain barrier and inflammatory response (65). Anti-inflammatory effects in ischemia/reperfusion model of the kidney with a pre- and post-treatment with EtP diminished NF-κB phosphorylation and reduced levels of High mobility group box 1 protein, Toll-like receptor (TLR)2 and TLR4, resulting in renal protection (66). In line with these findings, resuscitation with EtP markedly inhibited the phosphorylation of the p65 subunit, possibly thereby diminishing inflammation-inducing effects of TxT+H/R. In conclusion, during acute inflammation EtP may exert protective effects by reducing the systemic activation of inflammatory leukocytes ending in previously described diminished local tissue damage.

There are several limitations of our study that remain to be considered when interpreting the results. We investigated only one endpoint, which is 2 h post resuscitation, while clinical outcome with regard to systemic and local inflammation after trauma should be investigated in the later course. Furthermore, next to observed acute changes after TxT+H/R investigations of overall survival, organ failure and MOF require larger study groups and prolonged observational periods. While we included female LEWIS rats, based on recent literature, differences between age and gender should be considered as important confounding factors in the immune response to traumatic insult and post-traumatic outcomes, and thus those remain to be further investigated in future studies. Regarding the dose- and time-dependent effects of EtP, that have been demonstrated before, our study is limited to one applied dose (50 mg/kg). Specific dose- and time-dependent influence of EtP on the post-traumatic inflammation and outcomes have to be further evaluated. Furthermore, the findings should be correlated to histological evaluations, e.g., the effect of reduced CD11b but elevated CD62L expression in response to EtP treatment on neutrophil infiltration may be investigated by histology. Similarly, biochemical assays of oxidative damage would demonstrate the net effect of increased phagocyte activity and delayed apoptosis of neutrophils on tissues. Thus, the correlation of systemic modulations with local changes should be assessed in future studies.

## Data Availability Statement

The raw data supporting the conclusions of this article will be made available by the authors, without undue reservation.

## Ethics Statement

The animal study was reviewed and approved by the veterinary department of the regional council in Darmstadt, Germany (Hessen, Germany).

## Author Contributions

BR designed the study and obtained the grant. SD, NF, and NW performed the experiments. SD wrote the first draft of the manuscript. KK performed the histological evaluations. BR, NW, and AS performed the statistical analysis and revised the manuscript. AN, SE, MK, and IM made important intellectual contributions to the study. All authors contributed to the article and approved the submitted version.

## Conflict of Interest

The authors declare that the research was conducted in the absence of any commercial or financial relationships that could be construed as a potential conflict of interest.

## Abbreviations

APC: allophycocyanin
CD: cluster of differentiation
DAMP: damage-associated molecular patterns
EtP: ethyl pyruvate
FACS: fluorescence-activated cell sorter
fig.: figure
g: earth's gravitational acceleration
Gran^+^: RP-1 positive granulocytes
h: hour
H&E: hematoxylin-eosin
Hg: mercury
H/R: haemorrhagic shock with subsequent resuscitation
HS: haemorrhagic shock
IL: interleukin
kg: kilogram
LR: lactated Ringers solution
MABP: mean arterial blood pressure
MFU: mean fluorescence units
mg: milligram
min: minute
ml: milliliter
mm: millimeter
MOF: multiple organ failure
NF-κB: nuclear factor kappa-light-chain-enhancer of activated B-cells
*p*: *p*-value
PAMP: pathogen-associated molecular patterns
PBS: phosphate-buffered saline
PMNL: polymorphonuclear leukocytes
ROS: reactive oxygen species
RT: room temperature
sem: standard error of the mean
TLR: Toll-like receptor
TNF: tumor necrosis factor
TxT: blunt thoracic chest trauma
U: unit
°C: Celsius
%: percent.
